# Author Correction: Drug classification with a spectral barcode obtained with a smartphone Raman spectrometer

**DOI:** 10.1038/s41467-023-41386-4

**Published:** 2023-09-11

**Authors:** Un Jeong Kim, Suyeon Lee, Hyochul Kim, Yeongeun Roh, Seungju Han, Hojung Kim, Yeonsang Park, Seokin Kim, Myung Jin Chung, Hyungbin Son, Hyuck Choo

**Affiliations:** 1 Metaphotonics TU, Samsung Advanced Institute of Technology, Suwon, Gyeonggi-do 16419 Republic of Korea; 2 Machine Learning TU, Samsung Advanced Institute of Technology, Suwon, Gyeonggi-do 16419 Republic of Korea; 3 Department of Physics, Chungnam National University, Daejeon, 34134 Korea; 4 Institute of Quantum Systems, Daejeon, 34134 Korea; 5 School of Integrative Engineering, Chung-Ang University, Seoul, 06974 Republic of Korea; 6 Department of Digital Health, Samsung Advanced Institute of Health Science, Sungkyunkwan University, Seoul, 06355 Korea; 7 Department of Radiology, Samsung Medical Center, Sungkyunkwan University, Seoul, 06355 Korea; 8 Department of Data Convergence and Future Medicine, Sungkyunkwan University School of Medicine, Suwon, Gyeonggi-do 16419 Korea; 9 Medical AI Research Center, Research Institute for Future Medicine, Samsung Medical Center, Seoul, 06351 Korea

**Keywords:** Electrical and electronic engineering, Imaging techniques, Raman spectroscopy

Correction to: *Nature Communications* 10.1038/s41467-023-40925-3, published online 29 August 2023

In this article, the affiliation details for Seokin Kim were incorrectly given as ‘Department of Digital Health, Samsung Advanced Institute of Health Science, Sungkyunkwan University, Seoul 06355, Korea’ but should have been ‘School of Integrative Engineering, Chung-Ang University, Seoul 06974, Republic of Korea’. The original article has been corrected.

