# Supplementary material for: Drug classification with a spectral barcode obtained with a smartphone Raman spectrometer
Source: Nat Commun. 2023 Aug 29;14:5262. doi: 10.1038/s41467-023-40925-3 (PMC10465478; doi:10.1038/s41467-023-40925-3)
Supplement: Supplementary file 1 — Suppementary information [file 41467_2023_40925_MOESM1_ESM.pdf]

# Supporting information

## Drug Classification with a Spectral Barcode Obtained with a Smartphone Raman Spectrometer

*Un Jeong Kim[1], Suyeon Lee[1], Hyochul Kim[1], Yeongeun Roh[1], Seungju Han[2], Hojung Kim[1], Yeonsang Park[3,4], Seokin Kim[8], Myung Jin Chung[5,6,7], Hyungbin Son[8], Hyuck Choo[1]**

Contents

Supplementary Figure 1. Photograph and internal optics of the Raman module.
Supplementary Figure 2. Structure of DBRs and its optical characteristics.
Supplementary Figure 3. Unique spectral barcode of drugs from Raman fingerprints.
Supplementary Figure 4. Molecular structures of 11 chemical components of drugs.
Supplementary Figure 5. The list of 58 drugs and its RGB images.
Supplementary Figure 6. Reference Raman spectra of 54 drugs.
Supplementary Figure 7. Statistical analysis on extracted Raman spectrum of 42 Vitamin C Raman spectral barcodes.
Supplementary Figure 8. Confusion matrix for the brand name classification of the drug from the spectral barcode.
Supplementary Figure 9. Confusion matrix of the CNN classifying shape and color from the RGB image of the drugs.
Supplementary Figure 10. Confusion matrix of the combined CNNs identifying the brand name of the drugs from the spectral barcodes and RGB images.
Supplementary Table 1. Comparison of smartphone based spectrometers.

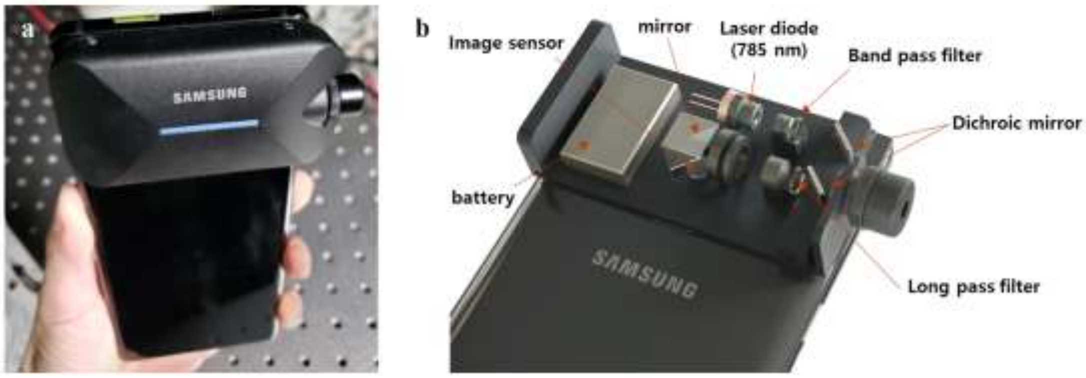


Supplementary Figure 1. **Photograph and internal optics of the Raman module. a** Photograph of the Raman module attached on the rear camera side of a Samsung Galaxy Note 9. **b** Internal optics and laser diode setup of the Raman module.

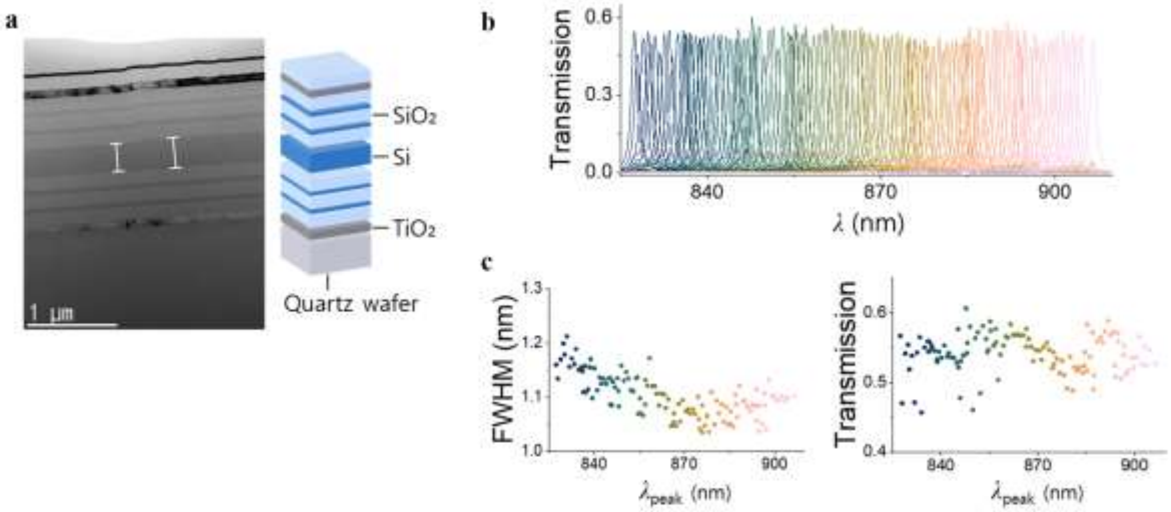


Supplementary Figure 2. **Structure of DBRs and its optical characteristics. a** Cross-section of the wavelength-selective band pass filter structure by transmission electron microscopy and schematics of the structure. The band pass filters consisted of a pair of DBRs separated with the Si layer, and the thickness of the Si layer tuned the transmission wavelength. **b** Series of transmission spectra of band pass filters. **c** FWHM and transmission of 120 filters as a function of the peak wavelength.

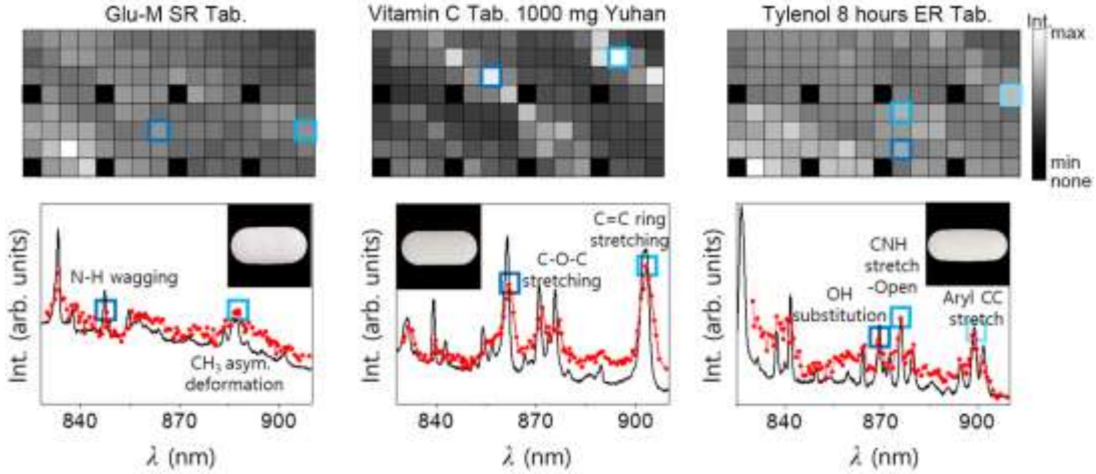


Supplementary Figure 3. **Unique spectral barcode of drugs from Raman fingerprints.** Spectral barcode of Glu-M SR for diabetes, Vitamin C, and Tylenol. Shown below are reference Raman spectra obtained with a commercial Raman spectrometer, and extracted Raman spectra from the spectral barcode, indicated by black solid line and red dots, respectively. Blue squares show two or three representative Raman bands of the spectral barcode and spectrum.

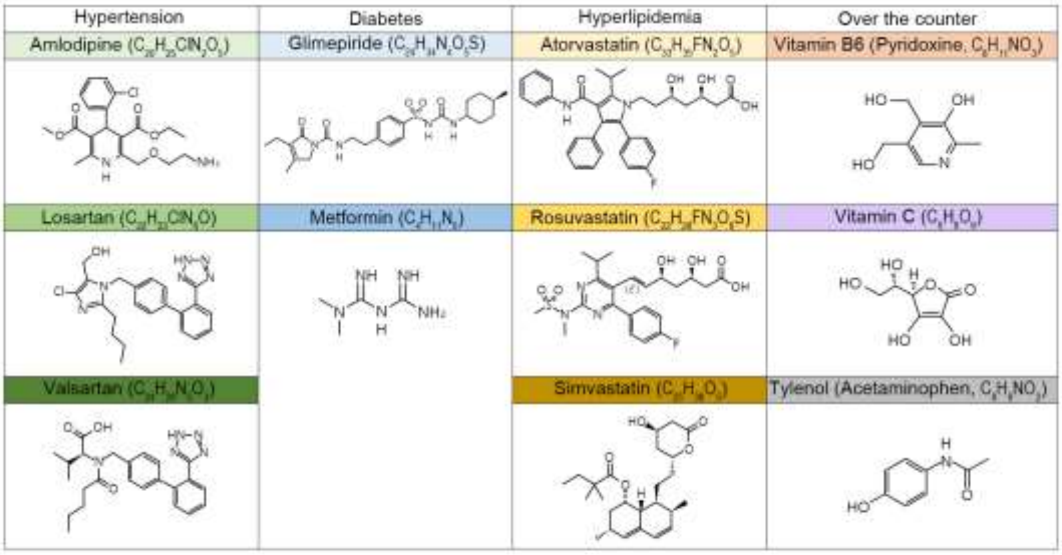


Supplementary Figure 4. **Molecular structures of 11 chemical components of drugs.** Molecular structure of major component of 11 categories of drugs: hypertension, diabetes, hyperlipidemia, and over-the-counter drugs.

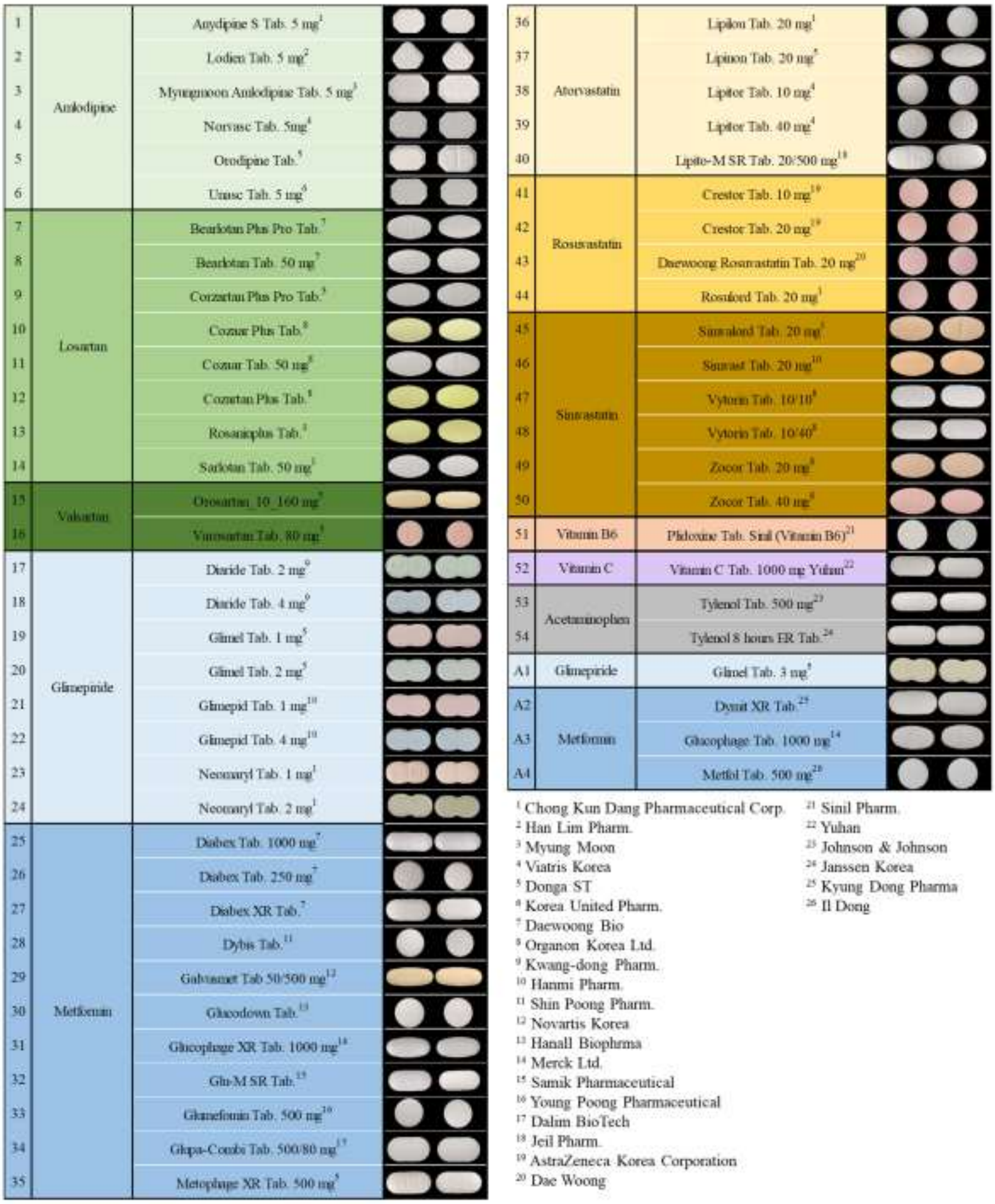


Supplementary Figure 5. **The list of 58 drugs and its RGB images.** Front and back appearance RGB images of 54 drugs obtained with a Galaxy Note 9 used for CNN training for RGB images and four untrained drugs, indicated by 1–54 and A1–A4, respectively. Only shape (snowman, circle, ellipse, and pentagon/octagon) and color (blue, yellow, green, white, and pink) information were used for recognition. The pharmaceutical company produced each drug is annotated.

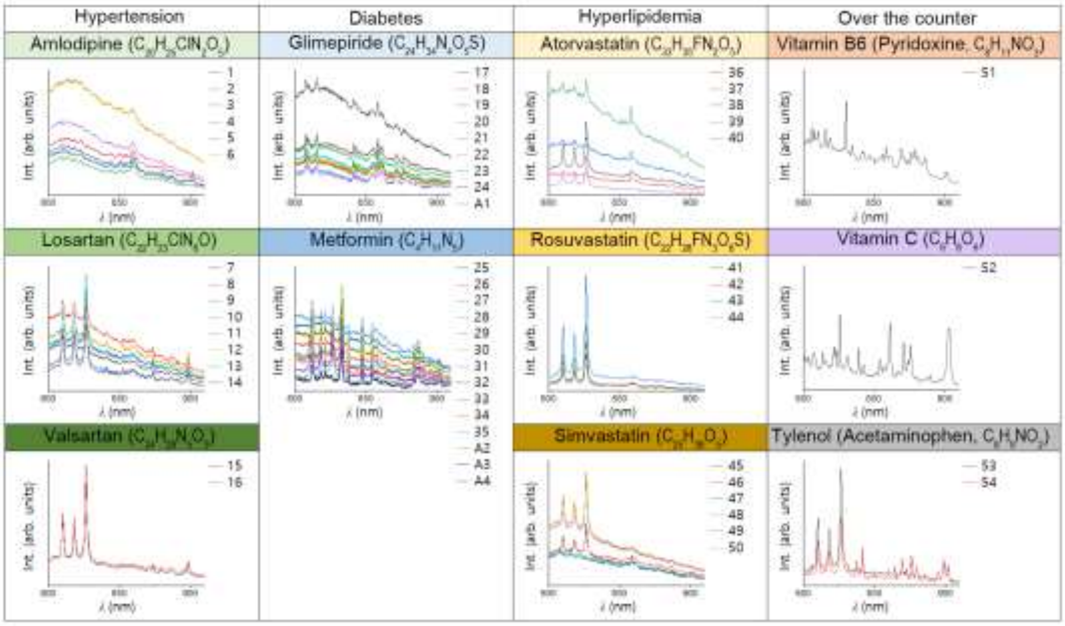


Supplementary Figure 6. **Reference Raman spectra of 54 drugs.** Reference Raman spectra, obtained with a commercial Raman spectrometer, of the drugs used for CNN training of the spectral barcode and the four untrained drugs; labeled as 1–54 and A1–A4, respectively. The drug name labeled by number can be found in Supplementary Fig. 5. All of the spectra are categorized by each major component. In the same category, the level of the background and additional peaks are due to additives.

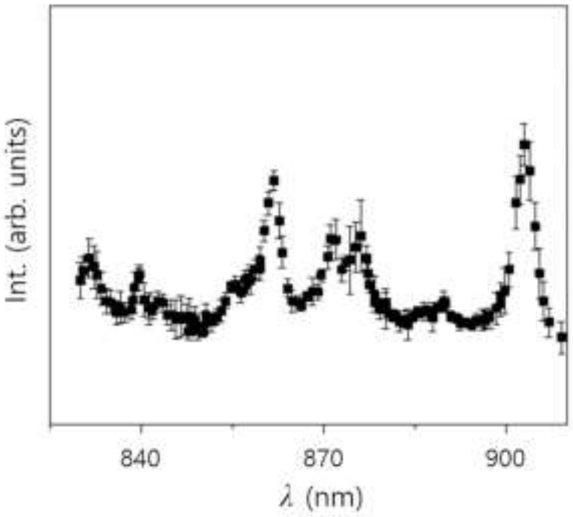


Supplementary Figure 7. **Statistical analysis on extracted Raman spectrum of 42 Vitamin C Raman spectral barcodes.** 42 Raman spectral barcodes of Vitamin C that were used for test of CNN are averaged and its error bar at each wavelength is displayed.

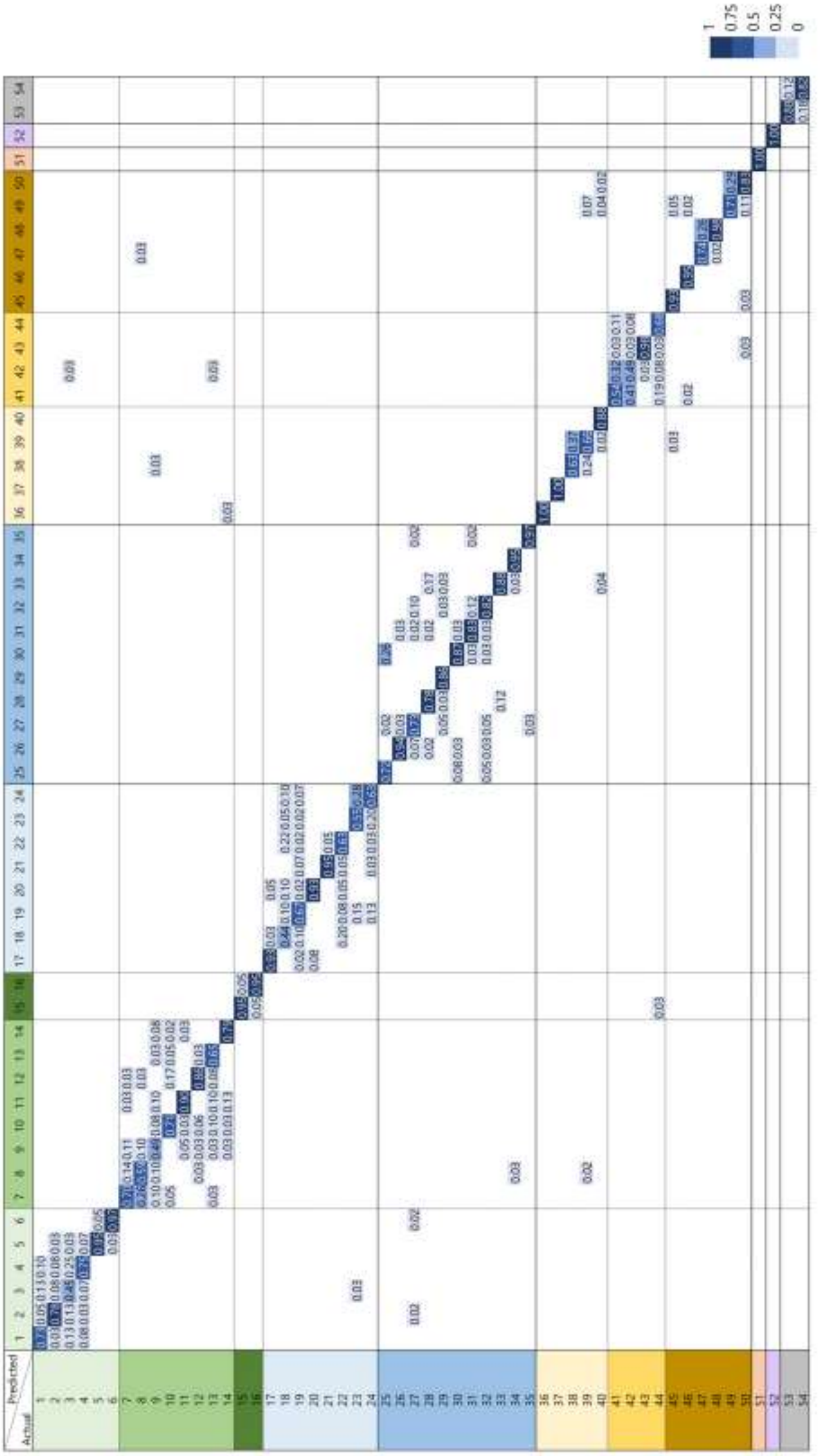

Supplementary Figure 8. **Confusion matrix for the brand name classification of the drug from the spectral barcode.** The drug name labeled by a number can be found in Supplementary Fig. 5.

| Actual \ Predicted | Amlodipine | Losartan | Valsartan | Glimepiride | Metformin | Atorvastatin | Rosuvastatin | Simvastatin | Vitamin B6 | Vitamin C | Tylenol |
|---|---|---|---|---|---|---|---|---|---|---|---|
| Amlodipine | 0.996 | | | | | | 0.004 | | | | |
| Losartan | | 0.987 | | | | 0.006 | 0.003 | 0.003 | | | |
| Valsartan | | | 1 | | | | | | | | |
| Glimepiride | 0.003 | | | 0.997 | | | | | | | |
| Metformin | 0.004 | 0.002 | | | 0.993 | | | | | | |
| Atorvastatin | | 0.005 | | | 0.01 | 0.957 | | 0.029 | | | |
| Rosuvastatin | | | 0.007 | | | | 0.993 | | | | |
| Simvastatin | | | | | | 0.004 | 0.008 | 0.988 | | | |
| Vitamin B6 | | | | | | | | | 1 | | |
| Vitamin C | | | | | | | | | | 1 | |
| Tylenol | | | | | | | | | | | 1 |

Supplementary Figure 9. **Confusion matrix of the CNN classifying shape and color from the RGB image of the drugs.**

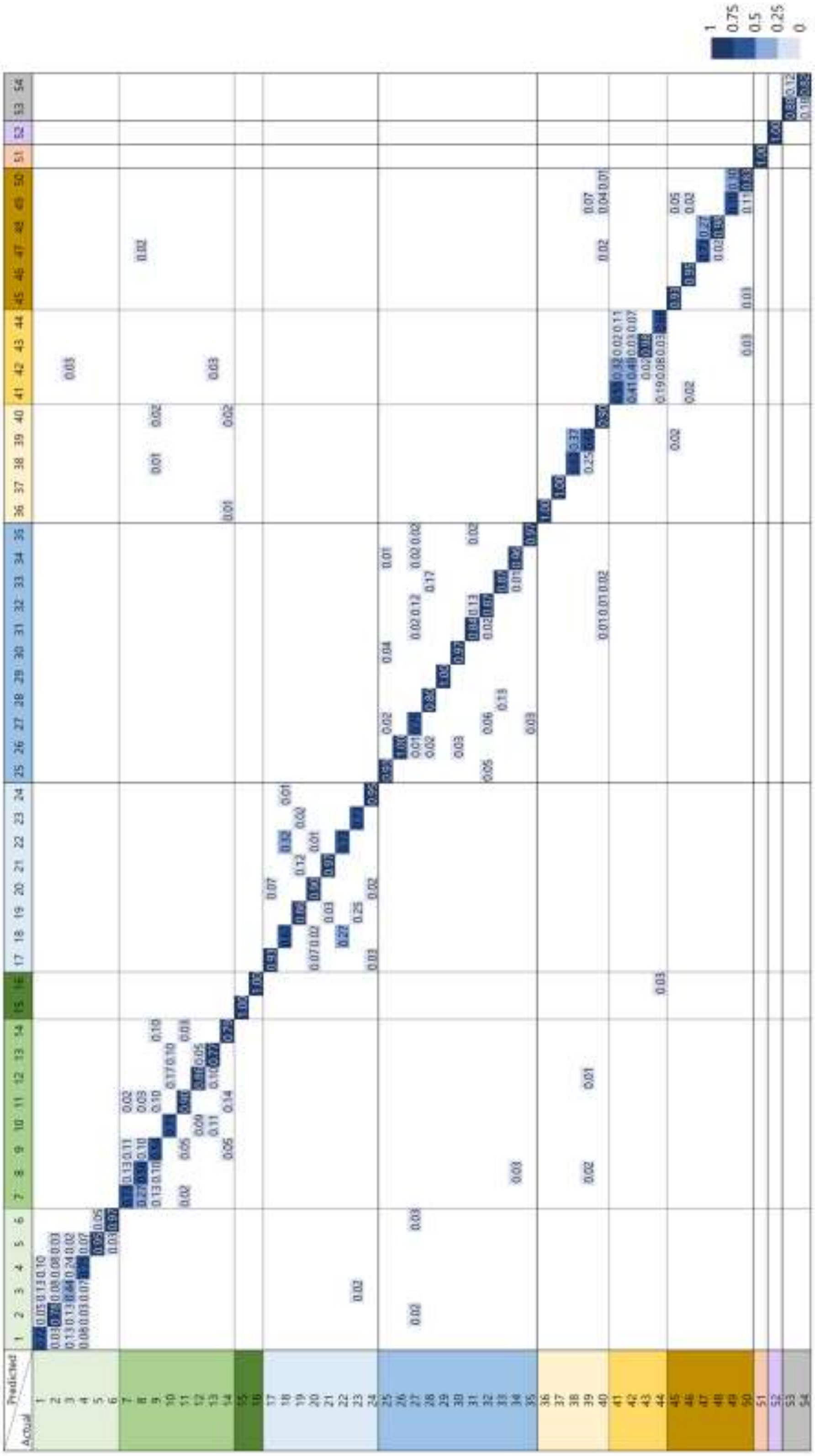

Supplementary Figure 10. **Confusion matrix of the combined CNNs identifying the brand name of the drugs from the spectral barcodes and RGB images.** The drug name labeled by a number can be found in Supplementary Fig. 5.

| | Spectral range | Spectral resolution | Form Factor | Optical Source | Detector | Spectrometer | Data analysis |
|---|---|---|---|---|---|---|---|
| CloudMinds's XI™ | 798~914 nm | < 1 nm | 159×79×27 mm (without case) | 785 nm LD | CMOS | External spectrometer | CNN based algorithm working at cloud |
| Changhong's H2 | near IR | - | - | - | CMOS | Imbedded spectrometer of Scio by Consumer Physics, Inc. | - |
| GoyaLab's IndiGo UV/VIS | 380~720 nm | <1.5 nm (FWHM) | 76×45×53 mm | - | CMOS | External spectrometer | - |
| Current work | 830~910 nm | 1~1.2 nm (FWHM) | 83×42×18 mm | 785 nm LD | CMOS | Image sensor based spectrometer | CNN based algorithm installed in On-device |

Supplementary Table 1. **Comparison of smartphone based spectrometers.** The works on smartphone based spectrometers demonstrate either the image sensor of the smartphone camera as detector, or smartphone as electronic controller and/or communication platform of external/installed spectrometer modules. One of the existing smartphone based Raman spectrometers, CloudMinds's XI™, is working by attaching external Raman spectrometer including the laser diode (LD) through the extra electronic control board to the smartphone. After taking Raman spectrum, the smartphone connects the cloud to analyze the data by deep learning algorithm. Changhong's H2 with a miniaturized and integrated material sensor was introduced at consumer electronics show in 2017. Material sensing near IR spectrometer (SCiO from Consumer physics, Inc.) was integrated in the smartphone (not image sensor of camera) in collaboration with Analog Devices Inc. Stand-alone miniaturized spectrometers with high spectral resolution which is controllable by android smartphones are commercially available, for example, Indigo UV/VIS from GoyaLab. In this work, the image sensor of the smartphone camera has been developed into spectrometers by forming Fabry-Perot filter arrays. Only external excitation and collection optics are needed to excite and collect Raman signals without additional connecting electronic board to the smartphone. This makes the smartphone spectrometer more compact with minimized external module and versatile. Higher spectral resolution can be acquired using grating type spectrometer, such as XI™, but still that of spectrometer in this work is high enough to measure Raman spectrum, producing lower data size which is favorable to data management in smartphone. Acquiring the spectrum of the objects can be done by the smartphone application, and further analysis can be done using CNN by on-device or connecting to the cloud. The spectral range of this work can be somewhat lower than that of previous works at this point, and this can be overcome by designing the wavelength set of filters and/or increasing number of channels in the array. There is a drawback of the developed spectrometer that changing the excitation wavelength for Raman measurement is limited due to the fixed set of filter wavelengths since the allowed wavelengths of excitation laser should be shorter than maximum wavelength of filters, respectively.
