# Supplementary material for: Drug classification with a spectral barcode obtained with a smartphone Raman spectrometer
Source: Nat Commun. 2023 Aug 29;14:5262. doi: 10.1038/s41467-023-40925-3 (PMC10465478; doi:10.1038/s41467-023-40925-3)
Supplement: Supplementary file 3 — Description of Additional Supplementary Files Document [file 41467_2023_40925_MOESM3_ESM.pdf]

<u>Description of Additional Supplementary Files</u>

File Name: Supplementary Movie 1

Description: **Demonstration of drug classification using Smartphone Raman spectrometer.** From measurement to obtaining classification results of three drugs (Tylenol, Lipito-M, Diabex) using galaxy note 9 integrated with 128CH band pass filter arrays on the image sensor of rear wide camera and using the pre-embedded CNN algorithm in the smartphone's AP. Raman signal was collected for 5 seconds.
